# Case Report: Based on the diamond theory, successful treatment of stubborn tibial nonunion after six surgeries using PRP-augmented therapy: a case report and literature review

**DOI:** 10.3389/fsurg.2025.1511722

**Published:** 2025-05-13

**Authors:** Shiheng Wang, Jiahua Wu, Zhihao Peng, Kunyu Wang, Zhihong Mo, Feng Wu, Jianrong Chen

**Affiliations:** ^1^ Department of Traumatic Orthopedics, The Eighth Clinical Medical College of Guangzhou University of Chinese Medicine, Foshan, Guangdong, China; ^2^ Department of Traumatic Orthopedics, Foshan Hospital of Traditional Chinese Medicine, Foshan, Guangdong, China; ^3^ Henan University of Chinese Medicine, Zhengzhou, Henan, China

**Keywords:** diamond theory, stubborn tibial fracture nonunion, fracture nonunion, platelet-rich plasma, case reports

## Abstract

**Background:**

The treatment of stubborn tibial nonunion remains a significant challenge. This case report describes a patient who underwent six surgeries and was treated using a stepwise surgical approach based on the diamond theory. The application of platelet-rich plasma (PRP) enhanced autologous iliac bone grafting combined with locked compression plate fixation ultimately achieved successful healing. This study also reviews relevant literature to explore the treatment experiences and outcomes of similar cases.

**Patient and methods:**

A middle-aged Asian male with severe heart disease underwent five failed surgeries, including two autologous bone graft procedures. Clinically, he presented with right calf pain, poor soft tissue condition on the anterior medial tibia, and signs of nonunion. During the sixth surgery, we applied a stepwise surgical procedure based on the diamond theory, achieving excellent clinical and bone healing, as well as satisfactory limb function at a follow-up of 11.3 months post-operation.

**Conclusion:**

We utilized a stepwise surgical procedure based on the diamond theory: locked compression plate internal fixation, PRP-enhanced structural autologous iliac bone grafting, and bioactive center creation techniques, providing a strong and stable mechanical and superior biological environment for the nonunion site.

## Introduction

Tibial fractures are among the most common types of fractures, with a high incidence of poor healing and nonunion, reported at an annual rate of 12%–19% (1). From a biological perspective, the cortical bone in the lower third of the tibia is relatively thick, while the amount of trabecular bone is minimal, resulting in a longer healing time for cortical bone. Additionally, the skin and soft tissue on the anteromedial aspect of the tibia are thinner, and the blood supply in the middle to lower third primarily relies on periosteal vessels after a fracture, leading to poor local blood supply and an increased risk of nonunion. The tibia bears the weight of the body and supports walking, and common clinical symptoms of nonunion include chronic pain, limping, joint instability, and weight-bearing restrictions. Literature indicates (1) that the incidence of nonunion in male tibial fractures is significantly higher than in females. Furthermore, other studies suggest (2) that conditions such as heart disease, smoking, and metabolic disorders significantly elevate the risk of fracture nonunion. Since commonly men bear the financial responsibility, tibial nonunion poses severe consequences for patients, their families, and society, especially in cases of persistent tibial shaft nonunion.

Currently, the established method for treating aseptic tibial nonunion is the implantation of autologous bone grafts (ABG) along with adequate stabilization (3, 4). This surgical approach is widely regarded as the gold standard for managing nonunion cases, including tibial nonunion (3, 4). In particular, autologous iliac bone grafting demonstrates excellent bone conduction, induction capacity, and bridging effect. However, relying solely on ABG to treat tibial nonunion may not yield satisfactory outcomes, even after multiple surgical interventions (3, 4). Consequently, an increasing number of clinicians are seeking more effective treatment strategies, especially for persistent tibial nonunion (3, 4). Over the past decade, with the introduction of the diamond theory of fracture healing by Giannoudis PV and others, clinical practices have diversified, leading surgeons to adopt various combination therapies for addressing nonunion. A retrospective study has reported (5) that the success rate of employing multiple treatment modalities for tibial nonunion, particularly in stubborn and complex cases, can be as high as 95%.

Currently, the literature (1) has reported several methods for treating aseptic tibial diaphyseal nonunion using a combination of internal or external fixation with bone grafting, most of which adhere to the diamond theory. These methods include, but are not limited to, intramedullary nail dynamization, replacement of intramedullary nails, supplemental plate augmentation, and external fixator bone transport. These techniques may involve grafting (such as autologous iliac bone grafting, bone marrow aspirate grafting, allograft, or synthetic materials). Among these approaches, the replacement of intramedullary nails is favored by many orthopedic surgeons and is often cited in the literature as the preferred method for treating aseptic diaphyseal nonunion (6). This technique is considered to have mechanical and biological advantages in most studies, yet its success rate varies significantly, ranging from 72% to 100%. However, it also has notable limitations, particularly insufficient bone grafting, making it unsuitable for segmental bone defects.

Another common technique is supplemental plate augmentation (7), which provides robust mechanical stability to the nonunion site and allows for adequate bone grafting and fixation. Nevertheless, this method incurs significant surgical trauma, is technically challenging, and may lead to complications such as deep infection or chronic pain, with a relatively low level of evidence (8–11). There is limited literature on converting static locked intramedullary nails to dynamic intramedullary nails for treating aseptic diaphyseal nonunion, and the reported success rates vary significantly (12).

The most representative method of external fixator bone transport is the Ilizarov technique, which is suitable for segmental bone defects and infected nonunion. Compared to plate fixation, this technique exerts less stress shielding and causes less damage to the periosteum and soft tissue, but it is technically demanding, has a long treatment duration, and the prolonged use of an external fixator may lead to potential infections (13). Additionally, some literature (13) has reported the use of compression plate fixation combined with vascularized or non-vascularized fibula grafting, which is applicable to segmental bone defects and offers excellent support and compressive strength. However, this method is technically complex, and the fibula graft requires an extended period of bone remodeling to meet weight-bearing demands, or else stress fractures may occur.

Some studies have also mentioned non-surgical treatment options for aseptic diaphyseal nonunion, such as electromagnetic field stimulation, extracorporeal shockwave therapy, and ultrasound-guided injections of growth factors [like PRP, bone morphogenetic proteins (BMPs), and bone marrow-derived mesenchymal stem cells (MSCs)]. These studies are mostly case reports or series, with low levels of evidence. In summary, bone grafting has become the “gold standard” in nonunion revision surgery, especially in treating atrophic and oligotrophic nonunions, necessitating the use of biological adjuncts to improve fixation (1).

Our treatment is unique in that it involved a middle-aged male patient who experienced six surgeries following a fracture of the lower third of the tibia, leading to four instances of nonunion (Table 1). Despite employing treatment methods recommended in the literature, the fracture remained unhealed. During the fourth revision surgery for nonunion, clinical examinations revealed that the patient had severe cardiac disease, along with symptoms of depression and anxiety. Due to the multiple previous surgeries, the iliac bone donor site was limited, and the condition of the soft tissue on the anteromedial aspect of the right tibia was extremely poor. Faced with this challenging reality, we urgently needed to heal this patient's stubborn tibial nonunion. We innovatively implemented a stepwise surgical approach based on the diamond theory: locked compression plate fixation, structural autologous iliac bone grafting, PRP-enriched grafts, and the creation of a biological chamber at the nonunion site. Following our surgical intervention, the patient has achieved satisfactory bone and clinical healing.

**Table 1:** Detailed information on each surgery performed on patients.

Number of surgeries	Surgery dates	Diagnosis	Types of nonunion	Surgical methods	Preoperative figures	Postoperative figures
First surgery	March 1, 2020	Tibial and fibular shaft fractures		LCP		Figures 1A,B
Second surgery	December 11, 2020	Tibial fracture nonunion with internal fixation failure	Hypertrophic	Double plate with AIBG	Figures 1A,B	Figures 1C,D
Third surgery	October 20, 2022	Tibial fracture nonunion	Hypertrophic	LCP with AIBG	Figures 1E,F	Figures 1G,H
Fourth surgery	March 31, 2023	Tibial fracture nonunion with internal fixation failure	Hypertrophic	UEF	Figures 1I,J	Figures 1K,L
Fifth surgery	June 15, 2023	Tibial fracture nonunion with infection and external fixator loosening	Oligotrophic	Removal of external fixator and VSD	Figures 1M,N	Figures 1O,P
Sixth surgery	September 23, 2023	Tibial fracture nonunion with internal fixation failure	Atrophic	PRP, LCP, AIBG	Figures 1O,P	Figures 1Q,R

LCP, locked compression plate; AIBG, autologous iliac bone grafting; VSD, vacuum sealing drainage; UEF, unilateral external fixator; PRP: platelet-rich plasma.

## Patients and methods

**Figure 1:**
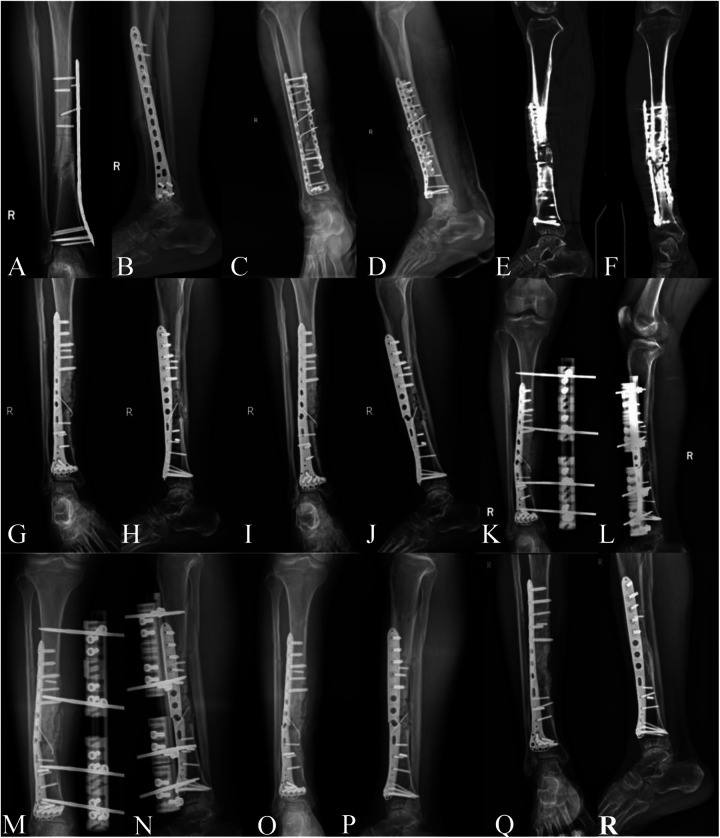
**(A,B)** Internal fixation failure and nonunion observed 9 months post-first surgery. **(C,D)** x-ray images on the day of the second surgery. **(E,F)** Preoperative CT showing bilateral plate failure and nonunion before the third surgery. **(G,H)** x-ray images on the day of the third surgery. **(I,J)** Preoperative x-ray images of the fourth surgery showing plate failure with external callus wrapping the fracture site, good alignment of the fracture. **(K,L)** x-ray images on the day of the fourth surgery. **(M,N)** Preoperative x-ray images for the fifth surgery. **(O,P)** Postoperative images after the fifth surgery, showing removal of external fixator, plate failure, and atrophic nonunion. **(Q,R)** 11.3 months post-sixth surgery, outpatient imaging shows indistinct fracture line, cortical bone bridge formation, and good tibiofibular alignment.

A 47-year-old Asian male with a BMI of 26.1 kg/m^2^ presented with a right tibiofibular fracture resulting from a fall on March 1, 2020. He underwent open reduction and internal fixation with a steel plate at another institution. Three months post-surgery, he began to walk without crutches against medical advice. Nine months after the surgery, it was discovered that the fracture had not healed, prompting him to seek treatment at our institution. The patient denies a history of open fractures, long-term smoking, and conditions such as diabetes, hypothyroidism, and cardiovascular diseases. Physical examination revealed pseudarthrosis at the fracture site, tenderness, and limping, consistent with clinical symptoms of nonunion (1). Imaging studies indicated a broken internal fixation device and hypertrophic nonunion (Figures 1A,B). The patient underwent his first revision surgery for nonunion on December 11, 2020 (Figures 1C,D), following a procedure reported in the literature (1): removal of the failed internal fixation, thorough debridement of fibrous scar tissue, sclerotic and necrotic bone at the fracture ends, reaming of the medullary canal, autologous iliac bone grafting, implantation of an appropriate plate with compression, and finally drainage and closure. A resorbable gelatin sponge of equal volume to the harvested iliac bone was placed in the iliac crest donor site for active hemostasis, and the incision was closed in layers. Postoperatively, standard orthopedic care was provided, and rehabilitation followed published protocols (14).

During a follow-up visit on October 17, 2022, the patient exhibited deformity in the right calf, and imaging revealed a broken internal fixation device and hypertrophic nonunion (Figures 1E,F). The second revision surgery differed from the first in that it utilized autologous iliac bone from the left side along with allograft bone; all other steps adhered to the previous protocol (Figures 1G,H). On March 27, 2023, the patient sustained another nonunion due to a fracture from a cycling accident, resulting in a repeat break of the internal fixation device (Figures 1I,J). In the third revision surgery, due to the poor soft tissue condition from the previous surgeries, imaging showed no significant displacement of the fracture ends, with continuous callus formation observed. Therefore, a unilateral external fixator was chosen for stabilization (Figures 1K,L), and the previously broken internal fixation device was not removed, with the other steps following the outlined procedures. On June 15, 2023, the external fixator was removed due to infection and loosening at the pin sites (Figures 1M,N). Subsequently, negative pressure drainage and a plaster splint were applied, along with aggressive antibiotic therapy to control the infection (Figures 1O,P). By September 23, 2023, after five prior surgical failures, the patient was psychologically and physiologically in urgent need of a successful sixth operation. Throughout all revision surgeries, pathological examinations and bacterial cultures were conducted, showing no signs of infection. All diagnoses of nonunion met the US Food and Drug Administration's (FDA) definition of nonunion. The definition of bone healing is the observation of at least three continuous cortical bone bridges or a blurred fracture line with continuous callus across the fracture line on a lateral x-ray of the lower leg. Clinical healing is defined as the absence of tenderness or pain on percussion at the fracture site, no abnormal motion at the fracture, and the ability to walk on level ground for 3 min without crutches. The main interventions for each revision surgery are detailed in Supplementary Table 1. Our case report strictly adhered to SCARE and CARE guidelines for case reporting (15, 16). This study has been approved by the Medical Ethics Committee of Foshan Traditional Chinese Medicine Hospital, approval number: KY [2023] 070. The patient information involved in this article has been obtained with the written informed consent of the patients themselves.

### Surgical procedure

After successful intrathecal anesthesia, the patient is placed in a supine position. The right lower limb and right iliac region are disinfected and draped. Cardiac function is closely monitored during the procedure.

#### Iliac Bone Harvesting

**Figure 2:**
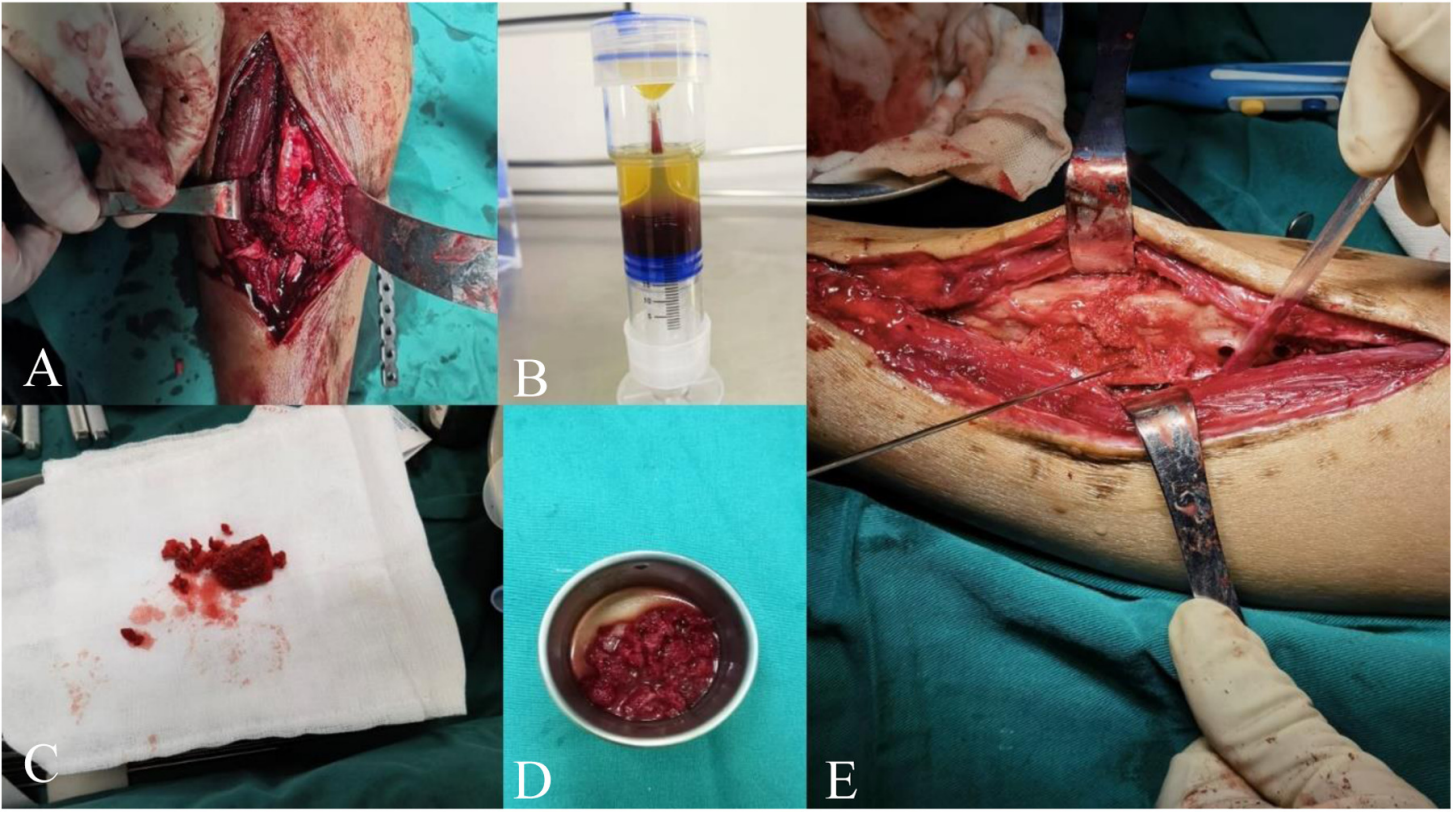
**(A)** Removal of non-viable tissue at the fracture site to freshen and roughen the bone surfaces, with a defect of approximately 1.5 cm. **(B)** Completed PRP kit preparation. **(C)** Transplanted iliac bone block and cancellous bone. **(D)** Transplanted bone block soaked in PRP solution. **(E)** PRP-saturated iliac bone block implanted into the defect.

**Figure 3:**
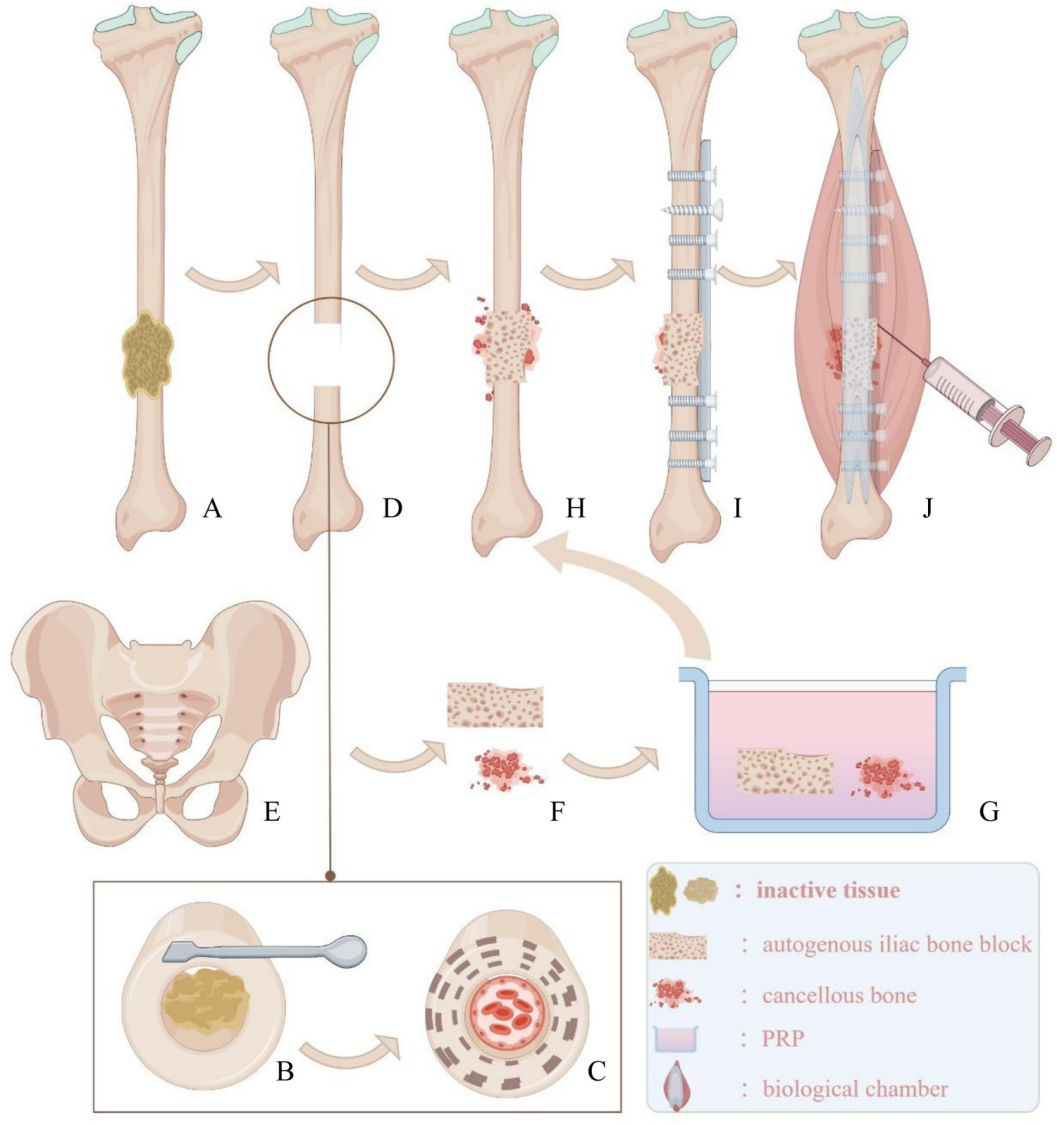
**(A)** Inactive tissue wrapped around the fracture end. **(B)** Use tools to remove inactive tissue. **(C)** Expose fresh and rough bone surfaces at the fracture end and reopen the medullary cavity. **(D)** Defect area for bone grafting. **(E,F)** Bone plate and cancellous bone from the ilium. **(G)** Soak the transplanted bone block in PRP solution. **(H)** Implant the bone block into the defect area. **(I)** Implant appropriate internal fixation. **(J)** Prepare a biological chamber at the fracture end and inject the remaining PRP solution into the capsule wall of the biological chamber using a syringe.

In the right anterior iliac crest area, bone is harvested. Depending on the size of the defect, an equal volume of cortical bone is chiselled, and some cancellous bone is also collected (Figure 2C, Figure 3E,F). The donor site is irrigated with 3°C 0.9% saline solution, and absorbable hemostatic gauze is used for packing. A gelatin sponge of the same volume as the harvested iliac bone is implanted, and the incision is sutured layer by layer.

#### Revisional Surgery for Nonunion

Step 1: Preparation of Bone Trench and Bone Bed:

A longitudinal incision is made along the original surgical scar on the right lower leg to expose the fracture ends. The failed internal fixation is removed, and all non-viable tissues (including fibrous tissue and sclerotic bone) at the non-union site are excised. A rough, fresh, hemorrhagic bone trench is obtained (Figures 2A, 3B,C,D), and the tissue and bone at the fracture ends are subjected to pathological examination.

Step 2: Preparation of PRP:

PRP is prepared using a standard protocol (17). Thirty milliliters of venous blood is drawn from the elbow, mixed with 3 ml of sodium citrate anticoagulant, and gently shaken to ensure thorough mixing. The blood is then centrifuged at 2,000 r/min for 10 min. After centrifugation, the PRP tube is left to stand for 5–10 min. Approximately 8 ml of PRP is aspirated with a 10 ml syringe and sent to the laboratory for counting. The PRP concentration is determined to be 909 × 10^9^/L after the count (Figure 2B).

Step 3: Fracture Reduction:

The surgical field is irrigated, and the alignment of the lower limb is corrected to ensure satisfactory bone position. Kirschner wires are used for temporary fixation. During the reaming process, 3°C 0.9% saline is dripped into the reaming site at a rate of 1 ml/s to prevent thermal injury.

Step 4: Preparation of PRP-Enhanced Bone Graft:

The iliac bone blocks are trimmed according to the size and shape of the defect. The trimmed iliac bone blocks and remaining cancellous bone are soaked in 8 ml of PRP solution for 10 minutes (Figures 2D, 3G). The PRP-enhanced iliac bone blocks are placed at the tibial bone defect, and the remaining cancellous bone mixed with PRP is used to fill the fracture site (Figures 2E, 3H). A locking compression plate (Aplus, China) is placed on the anterolateral side of the tibia, and appropriate screws are inserted to secure the plate under compression (Figure 3I). The bone is protected during reaming to prevent thermal damage. During surgery, high-frequency electrocautery is used for hemostasis, and sterile gauze and absorbable hemostatic gauze are applied for compression hemostasis.

Step 5: Preparation of Biologically Active Center:

After adequate hemostasis, the periosteum and the tissue around the fracture ends with good blood supply are tightly sutured. The remaining PRP solution is injected into the tissue surrounding the fracture site using a syringe (Figure 3J). The wound is closed layer by layer, and the skin is sutured. An elastic bandage is applied to secure the surgical site.

### Postoperative care and rehabilitation

During the surgery, intravenous second-generation cephalosporins are administered as prophylactic antibiotic treatment, continuing for 48 h postoperatively. The surgical site is dressed daily in a sterile manner until healing occurs. Ankle pump exercises are initiated the day after surgery, and weight-bearing time is determined based on imaging assessments. Postoperatively, the patient is prescribed oral vitamin D and calcium carbonate. Follow-up visits are scheduled at 1, 2, 3, 6, 9, and 12 months post-surgery. At each visit, imaging examinations are performed, and quality of life survey and pain assessments are completed. The imaging evaluation and survey data are collected by a medical graduate student and a senior trauma specialist. Any private or sensitive information related to the patient is anonymized. For cardiac care, enteric-coated aspirin is prescribed postoperatively, with close monitoring for potential drug-related bleeding.

## Results

At the 11.3-month follow-up after surgery, the patient, who suffered from severe coronary atherosclerosis and had undergone three unsuccessful revision surgeries for nonunion, achieved superior bony and clinical healing (Figures 1Q,R). There was no significant pain or tenderness at the nonunion site, no evidence of pseudarthrosis, and the patient has returned to their pre-injury lifestyle (Supplementary Figures 1A–C). No complications such as infection or chronic pain were observed in the iliac bone graft donor site. During each follow-up, we assessed the patient's pain, bone healing status, and quality of life using the Visual Analog Scale (VAS) for pain, Lane-Sandhu and Radiographic Union Score for Tibia (RUST) for bone healing assessment, and the Health Survey Short-Form 36 (SF-36) for quality of life (Table 2).

**Table 2:** Clinical and imaging outcomes of patients at different follow-up time points.

Variables	Preoperative	Follow-up 1 month	Follow-up 2 month	Follow-up 3 month	Follow-up 6 month	Follow-up 9 month	Follow-up 12 month
VAS	6	3	2	0	0	0	0
Lane-Sandhu	2	1	2	3	6	7	11
RUST	2	2	2	3	3	4	4
SF-36	37.60	42.70	53.86	61.87	71.92	80.39	88.65

SF-36, Health Survey Short-Form 36; RUST, Radiographic Union Score for Tibia; VAS, visual analogue scale.

## Discussion

Through our surgical procedure, this patient with a stubborn tibial nonunion achieved healing. Our technique differs from those reported in the literature by incorporating bone grafts enriched with PRP and creating a biological chamber at the nonunion site. In the study by Calori and Giannoudis, the concept of a “biological chamber” for fracture healing was introduced. We created such a “biological chamber” and additionally injected PRP into its walls to further enhance its biological activity. The biological chamber we developed forms the “highest biological activity center, the core of the diamond concept” (18). A study (19) used a combination of DBM (Decalcified Bone Matrix) and PRP for the treatment of bone defects, covering the surrounding tissue of the graft with tissue to protect the transplant and enhance the biological environment for bone regeneration. They also used PRP as a solution and mixed it with DBM powder to form a cement-like mixture, thereby improving biological activity and achieving a 93.75% healing rate. In our case report, this patient had previously undergone five surgeries, with poor biological environment and biological activity at the non-healing site. By soaking the transplanted bone blocks in PRP solution and creating an active chamber filled with PRP at the non-union site, we enhanced the biological activity of the “bone healing unit,” improving bone induction and proliferation. Ultimately, the patient achieved excellent clinical and bony healing. This finding is consistent with the studies by Trinchese et al. (20) and Bettega et al. (21), where the use of PRP enhanced the activity of the “bone healing unit” and resulted in good healing outcomes.

In a retrospective study of 186 cases, Wang et al. (14) created a 2 cm × 1 cm bone trench at each end of the femoral non-union site, implanted autologous iliac bone, and applied sufficient compression to the fracture ends using a steel plate. This resulted in excellent callus formation and a healing rate of up to 100%. Another key point of our technique is the preparation of a rough, fresh, hemorrhagic bone trench or bone bed for the transplanted bone blocks. This increases the friction between the fracture ends and the transplanted bone and, through compression techniques, improves the contact between the fracture ends and the graft, promoting intramedullary and extramedullary ossification and accelerating fracture healing. Our technique for preparing bone troughs or beds involves less bone deprivation, particularly in cases of atrophic nonunion, where extensive removal of the periosteum and bone can reduce the probability of healing (1). Our approach aims to maximize the biological activity at the fracture ends while maintaining a strong and stable foundation. In recent years, with the advent of regenerative medicine, the application of PRP has become increasingly widespread. PRP is an autologous blood product rich in concentrated platelets, containing platelet-derived growth factors (such as PDGF-aa, PDGF-bb, PDGF-ab), transforming growth factor β1 (TGF-β1), TGF-β2, vascular endothelial growth factor (VEGF), and epidermal growth factor (EGF). These growth factors enhance cell migration, proliferation, and differentiation, thereby promoting tissue regeneration (22). Research has shown that using adipose-derived stem cells combined with autologous PRP in canine bone defect models significantly promotes the proliferation and osteogenesis of bone marrow mesenchymal stem cells, facilitating the recovery of bone defects (23). Additionally, studies indicate that using PRP in conjunction with bone marrow mesenchymal stem cells for treating mid-shaft tibial defects in sheep results in significantly higher rates of new bone filling compared to control groups, as PRP promotes the proliferation and osteogenic differentiation of stem cells, accelerating the restoration and regeneration of bone and soft tissue (24, 25). In our report, the implanted iliac bone graft provided a structural scaffold and osteogenic cells. PRP contains abundant growth factors that can stimulate cell proliferation, differentiation, and angiogenesis. Meanwhile, the periosteum injected with PRP has a better synergistic osteogenic effect. Under the influence of this synergistic osteogenic effect, good bony union was finally achieved.

Memeo et al. (26) reported cases of using PRP combined with intramedullary nails to treat nonunion of forearm fractures, with all seven patients achieving healing; however, their technique was not detailed. We believe that their approach somewhat increased the biological activity at the nonunion site. In contrast, we not only injected PRP into the periosteum and adjacent soft tissues of the nonunion site but also soaked the transplanted bone graft in PRP solution, achieving better biological activity. Trinchese et al. (20) found improved bone proliferation effects when injecting a mixture of PRP and mesenchymal stem cells (MSCs) into the fracture gap. Bettega et al. (21) in an 18-case retrospective controlled study found that combining PRP with a small amount of autologous bone graft reduced the required bone graft by 60%. This finding aligns with Lee et al. (27), indicating that PRP enhances bone reconstruction in the early stages. The outcomes from our case confirm the findings reported in previous literature, and our report provides a standardized and novel method for applying PRP in fracture non-union revision surgery.

However, in another prospective randomized controlled study involving 120 cases (28), the clinical and radiographic efficacy of using recombinant human bone morphogenetic protein-7 (rhBMP-7) combined with PRP for treating long bone fracture nonunions was found to be inferior to that of the rhBMP-7 group. The suboptimal performance of the PRP group may be due to the lower concentration of growth factors extracted from PRP. In our study, the obtained PRP was laboratory-tested and showed a concentration of up to 909 × 10^9^/L, with most scholars agreeing that concentrations between 500 × 10^9^/L and 1,000 × 10^9^/L can significantly stimulate cell proliferation, migration, and collagen synthesis (29, 30). Furthermore, the aforementioned study (28) did not utilize autologous bone grafting, lacking a bone bridge and osteoconductive capability, which may also contribute to its subpar results. We not only used a higher dose and concentration of PRP but also employed autologous iliac bone grafting, regarded as the “gold standard,” ultimately achieving ideal results. This aligns with the findings of Lee (27) and Dohle (31), among others. Additionally, the PRP preparation technique they used (28) is relatively outdated, whereas the PRP preparation method has rapidly evolved over the past decade (17). In a unique case-control study (32), the healing rate of patients treated with PRP and external fixation (90%) was compared to the healing rate of a historical control group (85%), with no significant benefit from PRP observed. However, caution should be exercised in interpreting this result due to the relatively small sample size (*n* = 20) and the lack of statistical rigor.

In another prospective randomized controlled trial (33), results indicated that injecting PRP into the joint cavity during Pilon fracture surgery could reduce the risk of post-traumatic ankle arthritis, with significant reductions in IL-6, IL-8 and PGE2levels in the PRP group compared to controls. We believe that due to the patient's multiple surgical traumas, the soft tissue condition on the anteromedial side of the right tibia was extremely poor, gradually replaced by avascular fibrous scar tissue, significantly increasing the risk of soft tissue infection and necrosis. Therefore, injecting PRP into the anteromedial tissue may provide anti-inflammatory effects and promote soft tissue repair. Postoperatively, the patient did not experience wound drainage, infection, or necrosis, and healing was favorable (Supplementary Figures 1D,E). At the 14-month follow-up after surgery, physical examination revealed no significant tenderness at the fracture site and no evident motion at the pseudoarthrosis. The patient achieved a satisfactory gait and a good lower limb function score (The Lower Extremity Functional Scale (LEFS) score was 69 out of a possible 80 points).

Although PRP has shown significant effects in clinical treatment, as it has become a research hotspot, scholars continue to raise concerns about inconsistencies in PRP preparation techniques and quality. PRP with variable quality control not only affects treatment outcomes but also makes clinical results difficult to replicate (26, 34). Therefore, it is particularly important to prepare PRP with consistent quality assurance. Regarding the dosage of PRP, it is recommended to use at least 5 ml, as lower doses may be a potential factor for poor outcomes (17). If PRP is not used promptly after preparation and is stored for an extended period, it may undergo alterations and contamination (17). In addition, we do not place drainage devices in our technique. Although PRP has certain effects in promoting repair and anti-inflammation, there are also certain risks of hematoma formation and infection.

## Conclusion

Our method successfully treated a patient with severe coronary atherosclerosis who underwent six surgeries, including three failed revisions for fracture nonunion. In the fourth revision surgery, our approach achieved superior bony and clinical healing. Our technique not only provides strong stability at the fracture site but also maximizes biological activity at the fracture ends, further demonstrating the critical role of PRP in fracture healing. A limitation of our study is that it is based on a single case report, which cannot represent the diversity of the entire patient population. Another limitation is the lack of a control group, as comparisons with other therapies (such as intramedullary nail replacement, external fixation, or BMPs) were not conducted. More randomized controlled trials (RCTs) are needed to determine whether PRP is a more effective bone stimulator compared to BMPs and MSCs.

## Data Availability

The original contributions presented in the study are included in the article/Supplementary Material, further inquiries can be directed to the corresponding authors.
