# Supplementary material for: Case Report: Based on the diamond theory, successful treatment of stubborn tibial nonunion after six surgeries using PRP-augmented therapy: a case report and literature review
Source: Front Surg. 2025 May 13;12:1511722. doi: 10.3389/fsurg.2025.1511722 (PMC12106299; doi:10.3389/fsurg.2025.1511722)
Supplement: Supplementary file 1 [file Table1.docx]

Supplementary Table 1: Main Interventions for Each Revision Surgery

| Revision Frequency | Intervention Methods |
| --- | --- |
| First Revision Surgery | Anterolateral Locked Compression Plate, Anteromedial Reconstruction Plate, Right Autologous Iliac Bone Grafting |
| Second Revision Surgery | Anterolateral Locked Compression Plate, Left Autologous Iliac Bone Grafting, Allogeneic Bone Grafting |
| Third Revision Surgery | Unilateral External Fixator |
| Fourth Revision Surgery | Anterolateral Locked Compression Plate, Right Autologous Iliac Bone Grafting, Platelet-Rich Plasma |
