# Supplementary material for: Case Report: Based on the diamond theory, successful treatment of stubborn tibial nonunion after six surgeries using PRP-augmented therapy: a case report and literature review
Source: Front Surg. 2025 May 13;12:1511722. doi: 10.3389/fsurg.2025.1511722 (PMC12106299; doi:10.3389/fsurg.2025.1511722)
Supplement: Supplementary file 2 [file Supplementaryfile1.pdf]

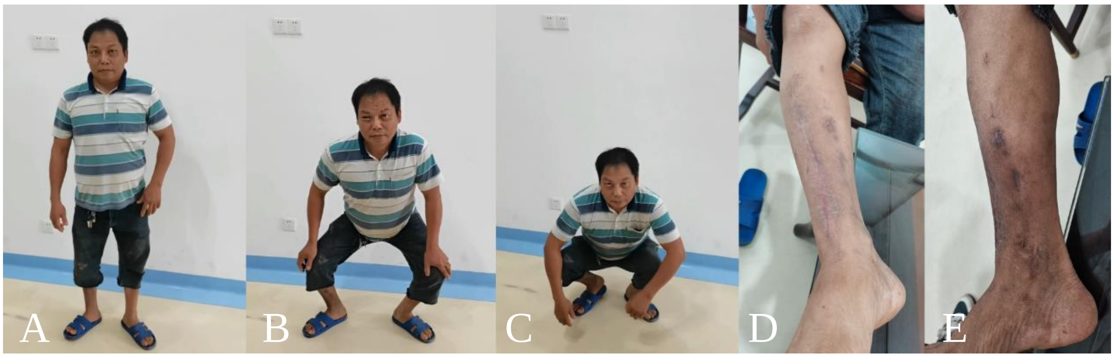

Supplementary Figure 1, ABC: September 2, 2024, outpatient follow-up shows good functional recovery of the patient's right calf. DE: September 2, 2024, outpatient follow-up shows good soft tissue healing in the affected limb, with no signs of redness, swelling, or infection.
